# Microglia limit the expansion of β-amyloid plaques in a mouse model of Alzheimer’s disease

**DOI:** 10.1186/s13024-017-0188-6

**Published:** 2017-06-12

**Authors:** Ruohe Zhao, Wanling Hu, Julia Tsai, Wei Li, Wen-Biao Gan

**Affiliations:** 1 Drug Discovery Center, Peking University Shenzhen Graduate School, Shenzhen, 518055 China; 2 Skirball Institute, New York University School of Medicine, New York, NY 10016 USA

**Keywords:** Alzheimer’s disease, Aβ plaque, Microglia depletion, Two-photon imaging, APP/PS1, CX_3_CR1^CreER/+^:R26^DTR/+^

## Abstract

**Background:**

Microglia are known as resident immune cells in the brain. β-amyloid (Aβ) plaques in the brain of Alzheimer’s disease (AD) are surrounded by microglia, but whether and how microglia affect the formation and maintenance of plaques remains controversial.

**Methods:**

We depleted microglia by injecting diphtheria toxin (DT) in *CX*_*3*_ *CR1* ^*CreER/+*^ *:R26* ^*DTR/+*^ (*CX*_*3*_ *CR1-iDTR*) mice crossed with *APPswe/PSEN1dE9* (*APP/PS1*) mice. Intravital time-lapse imaging was performed to examine changes in the number and size of Congo Red-labeled amyloid plaques over 1–2 weeks. We also examined spine density and shaft diameter of dendrites passing through plaques in a *PSAPP* mouse model of AD (*PS1*_M146L_ line 6.2 × Tg2576) crossed with *Thy1* YFP H-line mice.

**Results:**

We found that DT administration to *CX*_*3*_ *CR1-iDTR* mice efficiently ablated microglia within one week and that microglia repopulated in the second week after DT administration. Microglia depletion didn’t affect the number of amyloid plaques, but led to ~13% increase in the size of Aβ plaques within one week. Moreover, microglia repopulation was associated with the stabilization of plaque size during the second week. In addition, we found dendritic spine loss and shaft atrophy in the distal parts of dendrites passing through plaques.

**Conclusion:**

Our results demonstrate the important role of microglia in limiting the growth of Aβ plaques and plaque-associated disruption of neuronal connection.

**Electronic supplementary material:**

The online version of this article (doi:10.1186/s13024-017-0188-6) contains supplementary material, which is available to authorized users.

## Background

Accumulation of β-amyloid (Aβ) in the brain is a hallmark of Alzheimer’s disease (AD) [1, 2]. Studies of postmortem AD brains have revealed dystrophic neurites inside and surrounding Aβ plaques [3–6]. Studies in AD mouse models have also shown that Aβ deposition is associated with various neuronal abnormalities, including the formation of dystrophic neurites [5, 7, 8], dendritic spine loss [5, 7, 9, 10], synaptic dysfunction [11, 12] and abnormal neuronal firing [13, 14]. The variety of neuronal deficits associated with Aβ deposition likely contribute to memory loss and cognitive decline in AD patients [15–19].

Microglia are the resident immune cells in the central nervous system [20, 21]. They are clustered around amyloid plaques in both human AD brains [22, 23] and the brains of AD mouse models [24, 25]. Mutations in genes such as *CD33* and *TREM2* have been linked to microglia dysfunction and increased risks for AD [26–28]. Knockout of *CX*_*3*_ *CR1* [29, 30], *CD33* [27], or *TREM2* [31, 32] leads to altered plaque load, suggesting that microglia are involved in Aβ deposition. Activated microglia have been shown to phagocytose Aβ through scavenger receptor-mediated mechanisms [33–35]. Furthermore, in vivo imaging studies have shown that microglia volume surrounding plaques correlated with the decrease of plaque size [25]. Microglia processes enveloping plaque surface are suggested to constitute a barrier to prevent plaque expansion [36, 37], and the disruption of this barrier in *TREM2* deficiency mice is associated with the increase of dystrophic neurites [32, 37]. Together, these studies lead to a view that microglia are involved in the clearance of Aβ and limiting the expansion of plaques.

Although many lines of evidence suggest a role of microglia in restricting the growth of amyloid plaques, several studies have shown that microglia depletion does not affect the number and size of plaques in mouse models of AD. It has been reported that chronic microglia depletion for one month by activating suicide gene *HSVTK* under *CD11b* promoter has no effects on the formation or maintenance of plaques [38]. In another study, microglia are depleted by inhibiting colony-stimulating factor 1 receptor (CSF1R) with PLX3397 from Plexxikon. PLX3397 treatment for 4 weeks results in nearly complete depletion of microglia, but has no effects on plaque load in a mouse model of AD [39]. Thus, in contrast to the view that microglia are beneficial to limiting amyloid deposits, these depletion studies suggest that microglia have no significant effect on the formation and growth of amyloid plaques.

To better understand the role of microglia in Aβ deposition, we examined the number and size of plaques after depleting microglia by administration of diphtheria toxin (DT) to *CX*_*3*_ *CR1* ^*CreER/+*^ *:R26* ^*DTR/+*^ (*CX*_*3*_ *CR1-iDTR*) mice crossed with *APPswe/PSEN1dE9* (*APP/PS1*) mice. Using two-photon time-lapse imaging of Aβ plaques in mice more than 12 months old, we found that Aβ plaque size, but not plaque number, showed a significant increase over 7 days after microglia depletion. In addition, we found a significant reduction in dendritic spine density and dendritic diameter in the distal segments of dendrites passing through plaques. Taken together, our results suggest that microglia play an important role in limiting the growth of Aβ plaques and reducing neuronal damage associated with Aβ deposition.

## Methods

### Transgenic mice

*APPswe/PSEN1dE9* (*APP/PS1*) were purchased from Guangdong Medical Laboratory Animal Center, China. C57BL/6 mice expressing YFP in layer-V pyramidal neurons (1*Thy1* H-line) and *Rosa26-stop-iDTR* (*DTR*) mice were purchased from the Jackson Laboratories, US. *CX*_*3*_ *CR1* ^*CreER*^ mice were generated in New York University School of Medicine [40]. These mice were crossbred and litters were genotyped by PCR using the following primers: for *APP/PS1* mice, 5′- TCATGACTATCCTCCTGGTGG-3′ and 5′- CGTTATAGGTTTTAAACACTTCCCC-3′; for *CX*_*3*_ *CR1* ^*CreER*^ mice, 5′- AAGACTCACGTGGACCTGCT-3′, 5′- CGGTTATTCAACTTGCACCA-3′ and 5′- AGGATGTTG ACTTCCGAGTTG-3′; for *DTR* mice, 5′- CTGGCTTCTGAGGACCG-3′ and 5′-CGAAGAGTTTGTCCTCAACCG-3′. 12–24 months old quadruple transgenic mice were used for microglia depletion experiment and in vivo imaging. Age-matched animals with *APP/PS1* mutation but no *CreER* or *DTR* gene were used as controls.

For studies of dendritic abnormalities near plaques, mutant human amyloid precursor protein (FAD *APP*_670/671_ line Tg2576) and mutant human Presenilin 1 (*PS1*_M146L_ line 6.2) mice were obtained from Taconic and the University of South Florida, respectively. All experiments were done in accordance with the institutional guidelines.

### Labeling amyloid plaques in vivo

Mice were anesthetized with pentobarbital sodium (100 mg/kg) 24–48 h before imaging. Congo Red was injected into the subarachnoid space using a fine glass electrode guided by a micromanipulator. The electrode was backfilled with Congo Red (3 μl, 0.5% in artificial cerebral spinal fluid (ACSF), filtered through a 0.20 μm syringe filter before use, Sigma) and inserted through the thinned skull. After subarachnoid space was reached (as evidenced by free dye diffusion), Congo Red was then pressure injected with a picospritzer (20 p.s.i., 20 ms, 0.8 Hz) over 20–30 min. Dye labeling of amyloid plaques was typically observed within 30 min of injection.

### In vivo two-photon imaging of Aβ plaques

The procedure of transcranial two-photon imaging was described previously [7, 41]. Briefly, after exposing the skull surface and gluing the skull to a custom-made steel plate, a small region ~1 mm in diameter was thinned with a highspeed drill, and a microsurgical blade was then used to continue thinning until the skull area ~500 μm in diameter was ~20 μm in thickness. A picture of the brain vasculature in the thinned region was taken with a CCD camera and used as a landmark for future relocation. Animals were placed under a two-photon microscope, and image stacks of plaques within a depth of 300 μm from the pial surface were obtained in the step size of 1 μm with a 1.05 N.A. 25× water-immersion objective and with two-photon laser tuned to 880 nm. For each frame, a 512 × 512 pixel, zoom 1× image was taken from a 508 × 508 μm ROI. Image stacks from motor, somatosensory and visual cortices yielded a full three-dimensional data set of Aβ plaques labeled with Congo Red in these cortical regions. After imaging, the plate was gently detached from the skull and the scalp was sutured, and the animals were returned to their home cages until the next viewing.

### Microglia depletion

Depletion of microglia was performed according to previously published studies [40]. Animals received two doses of 10 mg of tamoxifen (20 mg/ml, dissolved in corn oil, Sigma) to induce Cre-mediated recombination and DTR expression, with a separation of 48 h between doses. Diphtheria toxin (Sigma) was diluted in PBS (50 μg/ml) and 1 μg of toxin was given i.p. for three consecutive days for depletion of microglia.

### Immunohistochemistry

Mice were anesthetized and perfused with 0.9% PBS. CNS tissue was removed and fixed in 4% PFA, rinsed with PBS and sectioned at 150 μm with a vibratome. Sections were permeabilized in 1% Titron X-100 in PBS for 3 h and blocked with 5% normal goat serum for 1 h. Sections were incubated overnight with primary antibodies against Iba1 (Wako, 1:500). Sections were then washed with PBS/0.05% Tween-20, and then incubated with Alexafluor-conjugated goat anti-rabbit IgG secondary antibodies (Life Technologies, 1:500) for 2 h. Sections were washed as before and mounted for imaging. Confocal images were obtained on a Biorad Radiance 2000 confocal microscope.

### Labeling and imaging fixed brain slice

Transversal slices (150–200 μm thick) of fixed brains were cut on a vibratome. Congo Red was used to visualized fibrillary amyloid in triple *YFP/APP/PS1* transgenic mice. Fixed brain slices were incubated in 0.5% Congo Red for 30 min and then rinsed with 0.1 M PBS. Slices were then mounted between two glass cover-slips in Vectashield (Vector Laboratories) and sealed with dental wax.

Labeled brain slices were imaged by laser scanning confocal microscopy using either a N.A. 1.25 40× or a N.A. 1.3 60× oil-immersion objective. Neuronal structures labeled with YFP and fibrillar amyloid deposits with Congo Red were scanned sequentially using 488 nm and 568 nm laser excitation, respectively. Image stacks at 0.3–1.0 μm steps were acquired to generate three-dimensional data sets of neuronal structures and amyloid plaques.

### Image analysis

To analyze the in vivo images of Aβ plaques, all images were processed using a custom-written Matlab algorithm. For each plaque, the frame with highest mean fluorescence intensity out of the entire image stack was identified as the center frame of this plaque. 2 additional frames above and below the center (5 frames combined) were used to analyze plaque size. In each image frame, plaque border was determined by setting a threshold of 3 times of standard deviation of the background fluorescence (mean value of the lowest 10% fluorescence intensity of the entire image) of the current frame. The averaged plaque area with the border was used as the plaque size.

To quantify fluorescence surrounding a plaque, fluorescence intensity across a radial line from the center point towards the outer area of a plaque was extracted. This radial line was drawn in a randomized direction and was kept in the same direction in analyzing the same plaque between different imaging sessions. This radial line was separated into the “inside” part and the “outside” part by the border of plaque. Fluorescence intensity across the line was normalized to a 0–100% scale with the highest value across the line as 100%. Normalized fluorescence intensity within 5 μm surrounding plaque border in the “outside” part was used as the measurement of fluorescence intensity surrounding the plaque.

To quantify changes of dendritic structures near Aβ deposits in fixed brain slices, dendritic spine densities and shaft diameters were measured with Metamorph software. Spine density and shaft diameter were measured for the proximal or distal dendritic segment (proximal or distal dendritic segments had comparable length, ranging from 20 to 80 μm), depending on the region in which the segment was located. Segments close to the soma and before the plaques were defined as proximal segments. Segments far away from the soma and after plaques were defined as distal segments. The border of Aβ deposits was determined as the position in which a sharp increase in fluorescence intensity was observed.

### Statistics

All data were presented as mean ± S.E.M. Kolmogorov-Smirnov test was used to test normality of sample’s distribution. Student’s t-test (two-tailed) was used to test for differences between groups whose distributions passed Kolmogorov-Smirnov test. Independent-samples Mann-Whitney test and related-samples Wilcoxon Signed rank test was used to compare differences between groups whose distributions did not pass Kolmogorov-Smirnov test. Significant levels were set at *P* ≤ 0.05. All statistical analyses were performed using the IBM SPSS Statistics 23.

## Result

### Cre-dependent microglia depletion in the brain

**Fig. 1:**
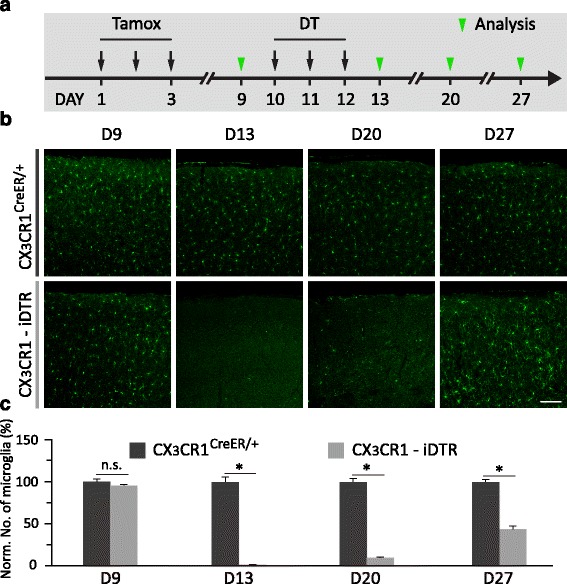
Microglia are depleted in *CX*_*3*_ *CR1-iDTR* mice over 1–7 days after DT administration and repopulate within 2 weeks after DT administration. **a**. Timeline of tamoxifen administration, DT administration and analysis of microglia number. Microglia were examined by Iba1 staining before (D9), 1 day after (D13), 1 week after (D20) and 2 weeks after DT administration (D27). **b**. Coronal sections of the cortex stained for Iba1 from *CX*_*3*_ *CR1* ^*CreER/+*^ and *CX*_*3*_ *CR1-iDTR* mice on D9, D13, D20 and D27, respectively. Scale bar, 100 μm. **c**. Quantification of Iba1 positive cells showing that the majority of microglia were depleted in the first week after DT administration and ~50% of microglia were repopulated during the second week in *CX*_*3*_ *CR1-iDTR* mice (*n* = 4, * *P* < 0.05, Mann-Whitney test)

Previous studies have reported that microglia depletion by activating suicide gene *HSVTK* or by inhibiting CSFR1 had no significant effect on the number and size of amyloid plaques in mouse models of AD [38, 39]. To better understand the role of microglia in amyloid deposition, we took advantage of a recently-developed method to deplete microglia by administrating diphtheria toxin (DT) into *CX*_*3*_ *CR1-iDTR* mice [40]. Two-month-old *CX*_*3*_ *CR1-iDTR* mice first received 10 mg of tamoxifen by gavage both on Day 1 and Day 3 to induce Cre-mediated recombination and DTR expression under *CX*_*3*_ *CR1* promoter. 1 μg DT was then administrated by intraperitoneal injections for three consecutive days on Day 10–12. The effect of DT administration on microglia depletion was examined 1 to 14 days after DT administration (Fig. 1a). We found that *CX*_*3*_ *CR1-iDTR* mice had a marked reduction of microglia 1 day after DT administration (Day 13) and the surviving microglia number was 0.8 ± 0.8% compared with control mice without DTR expression. Microglia remained largely absent in the cortex 7 days after DT administration (Day 20, 13.1 ± 1.3%). 2 weeks after DT administration, 57.4 ± 4.6% of microglia were found in the cortex of *CX*_*3*_ *CR1-iDTR* mice, indicating microglia repopulation occurred at this stage (Fig. 1b, c). Similar dynamics of microglia depletion and repopulation following DT administration were observed in the cortex of *APP/PS1/CX*_*3*_ *CR1-iDTR* mice at 15 month of age (Additional file 1: Figure S1). Thus, consistent with previous findings [40], these results show that after DT administration, microglia are efficiently depleted from the cortex of *CX*_*3*_ *CR1-iDTR* mice or *APP/PS1/CX*_*3*_ *CR1-iDTR* mice within one week, but start to repopulate during the second week.

### Microglia depletion over 2 weeks has no effect on the number of plaques

**Fig. 2:**
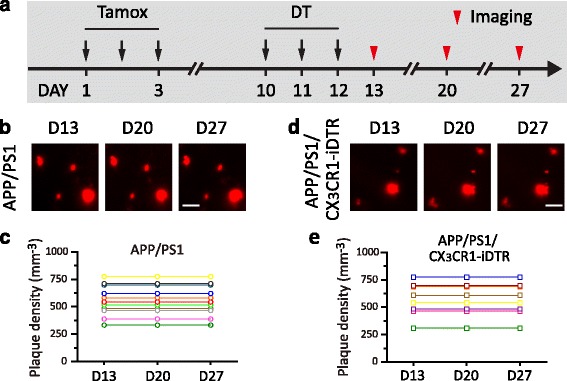
Ablation of microglia by DT administration has no effect on the number of amyloid plaques. **a**. Timeline of tamoxifen administration, DT administration and in vivo imaging of Congo Red-labeled plaques. Time-lapse imaging was performed 1 day after (D13), 1 week after (D20) and 2 weeks after DT administration (D27). **b**. Images of the same ROIs in *APP/PS1* mice over 1–2 weeks. **c**. Quantification of plaque density in *APP/PS1* mice in each ROI. **d**. Images of the same ROIs in *APP/PS1/CX*_3_ *CR1-iDTR* mice over 1–2 weeks. Neither elimination of existing Aβ plaques nor formation of new plaques occurred in *APP/PS1* or *APP/PS1/CX*_3_ *CR1-iDTR* mice. **e**. Quantification of plaque density in *APP/PS1/CX*_*3*_ *CR1-iDTR* mice in each ROI

To investigate whether microglia depletion affects Aβ deposition, we crossed *APP/PS1* mice with *CX*_*3*_ *CR1-iDTR* mice and depleted microglia in mice more than 12 months old (17 ± 2 months old). We first examined the effect of microglia depletion on the number of amyloid plaques over 1–2 weeks after DT administration (Fig. 2a). To label Aβ plaques, Congo Red was injected into the subarachnoid space 24–48 h before each imaging session (see Methods). Congo Red-labeled Aβ plaques were imaged through thinned-skull window using two-photon microscopy. Time-lapse imaging of the same cortical regions was performed 1 day (Day 13), 1 week (Day 20) and 2 weeks (Day 27) after DT administration (Fig. 2b, d). We found that the average plaque density in *APP/PS1/CX*_*3*_ *CR1-iDTR* mice before DT administration was 515 ± 63 mm^−3^, comparable to that in *APP/PS1* mice (574 ± 42 mm^-3^, *P* > 0.05). Consistent with the previous study showing no formation of new plaques over weeks in *APP/PS1* mice older than 9 months [42], we found that in >12 month-old *APP/PS1* mice, no disappearance of existing plaques or appearance of new plaques was detected over 1–2 weeks (Fig. 2b, c). Notably, the number of Aβ plaques also remained the same over 1–2 weeks in mice when microglia were depleted by DT administration (Fig. 2d, e). No new plaques or disappearance of existing plaques was observed during this period. Thus, consistent with the previous study reporting microglia depletion has no significant effect on the number of plaques [38, 39], our findings suggest that ablation of microglia by DT administration does not affect the number of Aβ plaques over 1–2 weeks.

### Microglia depletion leads to enlargement of Aβ plaques

**Fig. 3:**
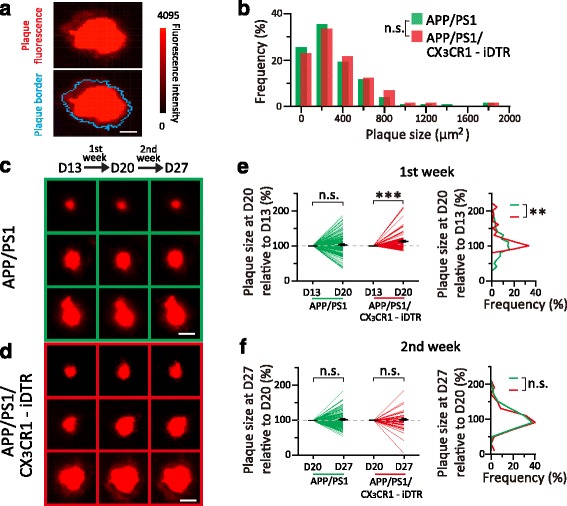
Microglia depletion induces increase in plaque size. **a**. *Blue curve* in the bottom panel indicates the identified plaque border for the example in the upper panel. The border of amyloid plaque was determined such that the fluorescence intensity of amyloid plaques was above 3 times of s.d. **b**. Distribution of plaque size on Day 13 in the cortex of *APP/PS1* mice (*green bars*, *n* = 129 plaques from 5 mice) and *APP/PS1/CX*_*3*_ *CR1-iDTR* mice (*red bars*, *n* = 75 plaques from 4 mice) (*P* = 0.23, Mann-Whitney test). **c**. Three examples of plaques imaged over 1–2 weeks in *APP/PS1* mice. **d**. Three examples of plaques imaged over 1–2 week in *APP/PS1/CX*_*3*_ *CR1-iDTR* mice. **e**. Change of plaque size in the first week after DT administration. No significant change in plaque size was detected within one week in *APP/PS1* mice (*n* = 129 plaques from 5 mice, *P* = 0.16, Wilcoxon matched-pairs signed rank test). Microglia depletion induced a significant increase in plaque size in *APP/PS1/CX*_*3*_ *CR1-iDTR* mice (*n* = 75 plaques from 4 mice, ***P < 0.001, Wilcoxon matched-pairs signed rank test). The distribution of plaque size at D20 relative to D13 is significantly different between these two groups (right panel, ** *P* < 0.01, Mann-Whitney test). **f**. Change of plaque size in the second week after DT administration. No significant change in plaque size was detected in *APP/PS1* mice (*n* = 92 plaques from 5 mice, *P* = 0.52, Wilcoxon matched-pairs signed rank test). Aβ plaques in *APP/PS1/CX*_*3*_ *CR1-iDTR* mouse cortex didn’t continue to grow from D20 to D27 (*n *= 40 plaques from 4 mice, *P* = 0.32, Wilcoxon matched-pairs signed rank test). The distribution of plaque size at D27 relative to D20 is not significantly different between these two groups (right panel, *P* = 0.52, Mann-Whitney test). Scale bar in a, c and d, 20 μm

In addition to examining the effect of microglia depletion on plaque number, we also compared the size of plaques over time in mice with or without microglia by two-photon time-lapse imaging. The Congo Red-labeled area of plaque was used as the measurements of plaque size. The border of amyloid plaques was determined such that the fluorescence intensity of amyloid plaques was above 3 times of standard deviation of the background fluorescence (Fig. 3a, see Methods). The size of Aβ plaques identified in *APP/PS1* (308.9 ± 27.6 μm^2^) and *APP/PS1/CX*_*3*_ *CR1-iDTR* mice (343.3 ± 35.5 μm^2^) were comparable before DT administration (*P* = 0.23, Fig. 3b). Consistent with previous studies [42–44], we found that the size of plaques showed no significant change over 1–2 weeks in *APP/PS1* mice older than 12 months (Fig. 3c–f). In contrast to the stable size of amyloid plaques in *APP/PS1* mice, we found that after DT administration plaques showed a significant increase (12.8 ± 3.2%, *P* < 0.001) in size from Day 13 to Day 20 in *APP/PS1/CX*_*3*_ *CR1-iDTR* mice (Fig. 3d, e). Furthermore, during the second week after DT administration when microglia repopulated (Fig. 1b, c), we found that *APP/PS1/CX*_*3*_ *CR1-iDTR* mice showed no additional increase of plaque size from Day 20 to Day 27 (*P* = 0.32, Fig. 3d, f). Taken together, these results strongly suggest that microglia have an important role in limiting the growth of amyloid plaques.

**Fig. 4:**
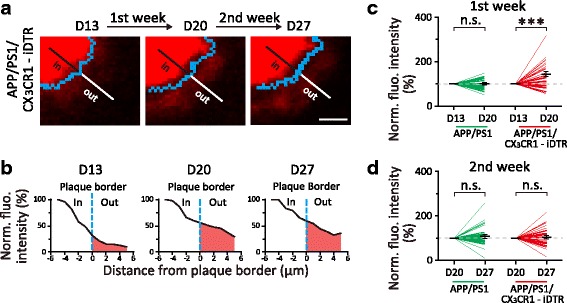
Absence of microglia induces increase of Congo Red-labeled Aβ fluorescence surrounding plaques. **a**. An example of an Aβ plaque in *APP/PS1/CX*_*3*_ *CR1-iDTR* mice at D13, D20 and D27, with blue curves indicating plaque border. A radial line from the center point towards the outer area of a plaque is separated by the plaque border, with the inside part representing area in the plaque (“in”, black) and the outside part representing the area surrounding the plaque (“out”, white). Scale bar, 5 μm. **b**. Fluorescence intensity across the line in a, with 5 μm inside the plaque and 5 μm outside the plaque. Shadow area represents fluorescence intensity surrounding plaques. **c**. Normalized fluorescence intensity surrounding plaques from D13 to D20. Microglia depletion in *APP/PS1/CX*_*3*_ *CR1-iDTR* mice induced a significant increase of fluorescence in the area surrounding plaque (*n *= 30 plaques, ***P < 0.001, Wilcoxon matched-pairs signed rank test). **d**. In the second week after DT administration (from D20 to D27), fluorescence surrounding plaques showed no additional increase in *APP/PS1/CX*_*3*_ *CR1-iDTR* mice (*n* = 30 plaques, *P* = 0.84, Wilcoxon matched-pairs signed rank test)

To further understand how the absence of microglia affects amyloid deposition, we examined Congo Red fluorescence intensity surrounding plaques. Fluorescence intensity across a radial line from plaque center towards outer area was measured (Fig. 4a) and separated into the “inside” part and the “outside” part by the border of plaque (Fig. 4b, see Methods). By examining fluorescence intensity within 5 μm surrounding plaque border in the “outside” part, we found a significant increase of Congo Red fluorescence intensity from Day 13 to Day 20 (43.7%, *P* < 0.001, Fig. 4c). In the following second week when microglia repopulated (Day 20 to Day 27), fluorescence intensity in the surrounding area didn’t continue to increase (*P* = 0.84, Fig. 4d). The change and stabilization of Congo Red fluorescence surrounding plaques after microglia depletion and repopulation provide further evidence that microglia are involved in limiting plaque growth.

### Aβ deposits cause abnormalities in dendrites passing through plaques

**Fig. 5:**
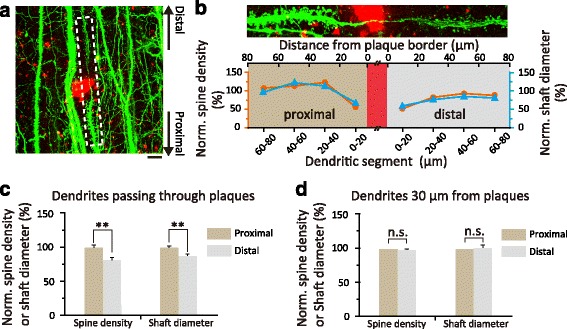
Aβ plaques cause spine loss and shaft atrophy in the distal segments of dendrites passing through plaques **a**. Confocal image of YFP labeled dendrites passing through and near Aβ plaques in the fixed brain slice of *PSAPP* mice crossed with *Thy1* YFP H-line mice. Aβ plaques were labeled with Congo red (*red*). A yellow fluorescent protein-labeled dendrite (*green*) showed a significant reduction in spine density and dendritic diameter after entering the plaque. Scale bar, 20 μm. **b**. Upper panel shows the dendritic branch passing through the plaque in dashed area in a. Bottom panel shows the quantification of spine density (*orange curve*) and shaft diameter (*blue curve*) in every 20 μm long of dendritic segments, ranging from 0 to 80 μm from plaque border. **c**. Distal segments of dendrites passing through Aβ plaques showed reduced spine density and shaft diameter compared to proximal segments. (*n *= 22 dendrites from 4 mice, **P < 0.01, student t-test). **d**. Spine density and shaft diameter of dendrites located greater than 30 μm away from plaque deposits showed no difference in the proximal and distal segments (*n *= 22 dendrites from 4 mice, *P* > 0.05, student t-test)

Our findings in *APP/PS1/CX*_*3*_ *CR1-iDTR* mice showed that microglia depletion resulted in ~13% increase in plaque size within one week. Based on the averaged plaque size in *APP/PS1* mice (308.9 ± 27.6 μm^2^, Fig. 3b), we estimated that for each plaque the cortex area covered by Aβ accumulation increase by ~40 μm^2^ over one week in the absence of microglia. Previous studies have shown various abnormalities of axons and dendrites inside or near plaques [5, 7–10]. It is possible that ~13% expansion of plaques after microglia depletion may lead to more extensive damage in neuronal circuits. To better understand neuronal damage associated with plaques, we examined spine density and shaft diameter of dendrites passing through and outside plaques in fixed brain slices. In this experiment, *PS1* mutant mice (*PS1*_M146L_ line 6.2) were first crossed with *APP* mutant mice (FAD *APP*_670/671_ line Tg2576). This mouse model of AD (*PSAPP* mice) was further crossed with *Thy1* YFP H-line mice to visualize dendrites and dendritic spines of pyramidal neurons in fixed brain slices from 6 to 10-month-old mice. Amyloid plaques were labeled with Congo Red (Fig. 5a). When dendritic segments passing through plaques were divided into “proximal” segments (segments between the cell body and the plaque) and “distal” segments (remaining segments after exiting the plaque, Fig. 5a, b), we found that distal segments of dendrites passing through plaques showed 19.4 ± 2.8% reduction in spine density and 11.1 ± 1.3% reduction in shaft diameter (*P* < 0.01) as compared to proximal segments (Fig. 5c). In contrast, dendrites that did not pass through plaques and were located > 30 μm away from plaque border showed no significant difference in spine density and shaft diameter between the corresponding distal and proximal segments (Fig. 5d). These findings show that amyloid plaque formation not only leads to dendritic abnormalities inside plaques, but also causes more global changes in distal segments of dendrites passing through plaques. The alterations of distal parts of dendrites passing through plaques suggest that ~13% expansion of amyloid plaques after microglia depletion could cause sizable damage in neuronal connectivity.

## Discussion

It is generally believed that microglia are involved in regulating Aβ deposition, a key hallmark of Alzheimer’s disease. However, the precise role of microglia in plaque formation and maintenance remains unclear. There are conflicting views on whether microglia are critical for limiting Aβ plaques, or microglia have no effect on plaque deposition. In this work, we examined the function of microglia in Aβ deposition by depleting microglia and imaging Aβ plaques in the living cortex of an *APP/PS1* mouse model. Our findings indicate the size of amyloid plaques increases in the absence of microglia. We further show that Aβ plaques are associated with dendritic spine loss and shaft atrophy in dendrites passing through plaques. These findings suggest that microglia play an important role in limiting Aβ plaques growth and neuronal damage in Alzheimer’s disease.

### Microglia limit the expansion of Aβ plaques

Mutations of *CD33* and *TREM2* genes expressed in microglia have been linked to increased risks of AD [26, 27, 37]. Altered Aβ load has been observed in *CX*_*3*_ *CR1* ^*−/−*^ [29, 30], *TREM2* ^−/−^ and *CD33* ^−/−^ mice [27]. In vitro studies have shown that microglia phagocytose Aβ [33–35]. Furthermore, in vivo studies have shown that Aβ is localized within microglial lysosomes and microglia volume surrounding plaques correlates with the reduction of plaque size over one month [25]. Together, these studies strongly suggest a role of microglia in Aβ clearance and/or regulating d Aβ deposition. However, previous studies of depleting microglia by introducing suicide gene *HSVTK* or inhibiting CSFR1 have shown that microglia depletion has no effect on both formation and maintenance of plaques [38, 39]. In the present study, using Cre-dependent microglia depletion and time-lapse imaging, we have now provided direct evidence that in the AD mouse model older than 12 months, microglia depletion over 1 week does not affect the formation or maintenance of amyloid plaques, but leads to a ~13% enlargement of plaque size in the cortex (Fig. 3d, e). Furthermore, 2 weeks after DT administration, microglia repopulation was associated with the stabilization of plaque size (Fig. 3d, f). Our findings strongly suggest the role of microglia in restricting the expansion of plaques.

It is important to point out several differences between previous studies and our work on the effect of microglia depletion. One difference is that previous studies examined the number and size of amyloid plaques using fixed brain tissues from different animals with or without microglia depletion [38, 39]. The variability in plaque number and size between different animals may make it difficult to detect relatively small changes in plaque size after microglia depletion. Taking advantage of time-lapse imaging, we have been able to track the same plaques over time and reveal the relative small changes (~13% over one week) of plaque size in response to microglia depletion. It is also important to note that microglia depletion in previous studies may take longer to occur than in our studies. The prolonged process of microglia depletion might cause compensatory responses such as astrocyte activation, which could lead to degradation of Aβ [45, 46]. Further studies are needed to address these possibilities in order to better understand the role of microglia in amyloid plaque deposition.

In addition to microglia in the brain, peripheral myeloid cells also express CX_3_CR1 and therefore could be depleted by DT administration in *APP/PS1/CX*_*3*_ *CR1-iDTR* mice. However, microglia and peripheral CX_3_CR1^+^ cells have substantially different turnover rates and are derived from different precursor populations [40, 47–50]. Microglia are long-lived population [47] and it has been shown that when tamoxifen is administrated ~30 days prior to the administration of DT, *CX*_*3*_ *CR1-iDTR* mice have a dramatic reduction of microglia within 1 day after DT administration [40]. On the other hand, CX_3_CR1^+^ cells in the spleen or blood are not affected as these peripheral CX_3_CR1^+^ myeloid cells in *CX*_*3*_ *CR1-iDTR* mice are replenished through a CX_3_CR1^−^ bone marrow precursor [47–50] and no l[nger express DT receptors 30 days after tamoxifen administration [40]. In our experiment, the time period between tamoxifen and DT administration was 7 days. Based on the rapid turnover of peripheral CX_3_CR1^+^ cells (Fig. 2 in ref. [40]), we expect that over this 7 day period the majority of peripheral CX_3_CR1^+^ cells would be replenished through a CX_3_CR1^−^ bone marrow precursor and do not express DT receptors. A small fraction of peripheral myeloid cells would still express DT receptors and would be depleted following DT administration. Therefore, we could not completely rule out the possibility that in addition to microglia depletion, the depletion of some peripheral CX_3_CR1^+^ myeloid cells may also affect the growth of amyloid plaques. Future studies to specifically deplete peripheral CX_3_CR1^+^ myeloid cells using bone marrow transplantation are needed to determine the potential contribution of peripheral CX_3_CR1^+^ cell population in amyloid plaque formation.

### Plaque growth may cause extensive neurite damage

A variety of studies have shown that abnormal spine density, spine turnover and diameter of dendritic shaft are associated with Aβ plaques [5, 7–9, 51]. In the current study, we found dendritic spine loss and shaft atrophy in the distal segments of dendrites passing through plaques when compared with proximal segments in the *PSAPP* mouse model of AD (Fig. 5a–c). This distal effect was not found in the dendrites which did not pass through plaques and were 30 μm away from Aβ plaques (Fig 5d). These results suggest that the expansion of Aβ plaques could cause damage not only in the local position but also to the entire distal segments of dendrites passing through. In this way, amyloid deposits might disrupt the signal propagation and protein transportation generated from soma and further lead to the degeneration of distal segments. The distal effect in dendrites passing through plaques also implies that extensive damage of neurites could be caused by enlargement of Aβ plaques when microglia are absent or microglia function is altered. These findings underscore the role of microglia in restricting the enlargement of plaques and limiting the neuronal damage in Alzheimer’s disease. The expansion of amyloid plaques after microglia depletion and plaque-associated dendritic abnormalities likely contribute to memory loss and cognitive decline in AD [15–19]. However, it is worth mentioning that microglia in AD mice also likely have impacts on the function of neuronal circuits and animal’s behaviors beyond their role in amyloid deposition and associated dystrophic neuritis [40, 52]. Thus, the depletion of microglia and resulting plaque expansion may or may not indicate disease worsening in AD mice. Future studies are needed to examine the impact of microglia depletion on neuronal network activity and plaque-associated synaptic abnormalities in the living mouse cortex in order to better understand microglia functions in AD. Furthermore, while plaque-associated dystrophic neurites have been shown in several AD mouse models and AD patients [4, 5, 7, 9, 51, 53], whether similar effects of amyloid plaques on distal dendrites observed in *PSAPP* mice also occur in other mouse models of AD remains to be investigated.

## Conclusion

Our data highlight that microglia play an important role in limiting the growth of Aβ plaques. Microglia depletion does not affect plaque numbers, but causes a significant increase of plaque size over one week. Furthermore, microglia repopulation is associated with the stabilization of plaque size. Restricting the growth of plaques by microglia could have a significant impact on reducing abnormalities in dendrites passing through amyloid plaques. Taken together, our studies reveal an important role of microglia in limiting the growth of Aβ deposition and neuronal damage in the pathogenesis of AD.

## Additional file

Additional file 1: Microglia are depleted in *APP/PS1/CX*_*3*_ *CR1-iDTR* mice over 1–7 days after DT administration and repopulate within 2 weeks after DT administration. **a**. Timeline of tamoxifen administration, DT administration and two-photon (2P) imaging. Microglia were examined before (D9), 1 day after (D13), 1 week after (D20) and 2 weeks after DT administration (D27). **b**. Time-lapse imaging in the same region in *APP/PS1/CX*_*3*_ *CR1-iDTR* mice and microglia morphology is revealed by CreER-IRES-EYFP under *CX*_*3*_ *CR1* promoter. The effects of DT administration over 1–2 weeks are similar to the effects on *CX*_*3*_ *CR1-iDTR* mice (Fig. 1b). (PDF 1380 kb)

## Abbreviations

ACSF: artificial cerebral spinal fluid
AD: Alzheimer’s disease
APP/PS1: APPswe/PSEN1dE9
Aβ: β-amyloid
CX_3_CR1-iDTR: CX_3_CR1^CreER/+^:R26^DTR/+^
DT: diphtheria toxin
DTR: diphtheria toxin receptor
PSAPP: PS1_M146L_ line 6.2 × Tg2576
ROI: region of interest
