# Supplementary figures and images for: Microglia limit the expansion of β-amyloid plaques in a mouse model of Alzheimer’s disease
Source: Mol Neurodegener. 2017 Jun 12;12:47. doi: 10.1186/s13024-017-0188-6 (PMC5468952; doi:10.1186/s13024-017-0188-6)

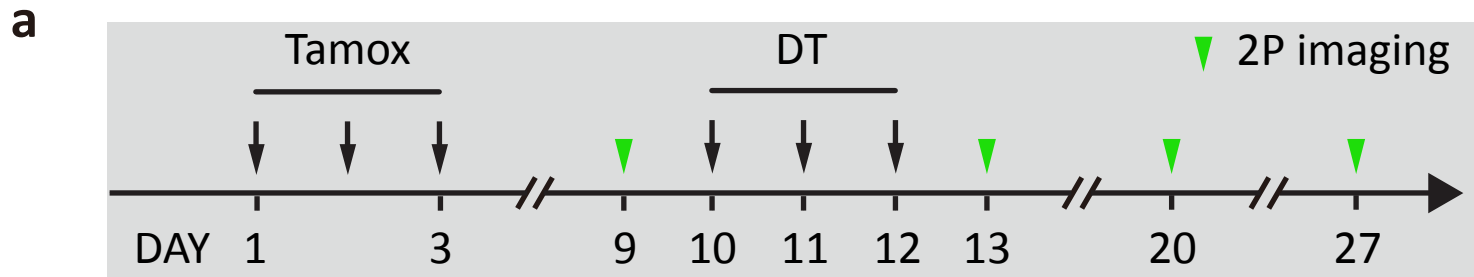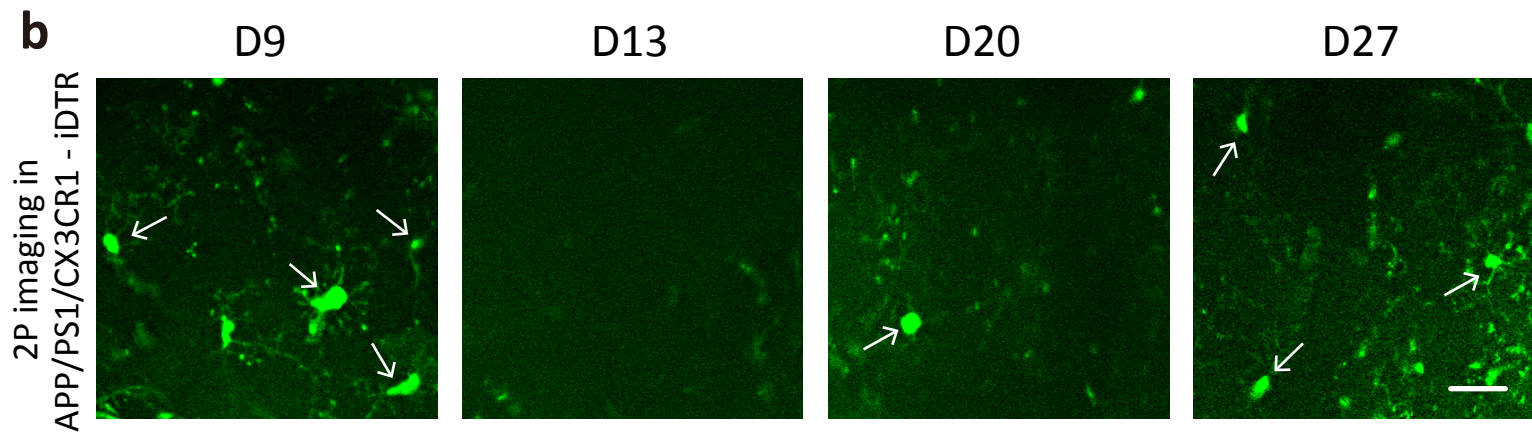

Supplement: Additional file 1: — Microglia are depleted in APP/PS1/CX 3 CR1-iDTR mice over 1–7 days after DT administration and repopulate within 2 weeks after DT administration. a. Timeline of tamoxifen administration, DT administration and two-photon (2P) imaging. Microglia were examined before (D9), 1 day after (D13), 1 week after (D20) and 2 weeks after DT administration (D27). b. Time-lapse imaging in the same region in APP/PS1/CX 3 CR1-iDTR mice and microglia morphology is revealed by CreER-IRES-EYFP under CX 3 CR1 promoter. The effects of DT administration over 1–2 weeks are similar to the effects on CX 3 CR1-iDTR mice (Fig. 1b). (PDF 1380 kb) [file 13024_2017_188_MOESM1_ESM.pdf]
